# Time to postpartum family planning initiation and its predictors among mothers coming for first measles vaccination at Family Guidance Association of Ethiopia, Dessie Model Clinic, Northeast Ethiopia: cox-regression model

**DOI:** 10.1186/s12978-023-01608-w

**Published:** 2023-04-29

**Authors:** Bezawit Adane, Elsabeth Addisu, Melaku Yalew, Yitayish Damtie, Mastewal Arefaynie, Segenet Zewdie, Yitbarek Wasihun, Bereket Kefale

**Affiliations:** 1 Department of Epidemiology and Biostatistics, School of Public Health, College of Medicine and Health Sciences, Injibara University, Injibara, Ethiopia; 2 Department of Reproductive and Family Health, School of Public Health, College of Medicine and Health Sciences, Wollo University, Dessie, Ethiopia; 3 Department of Pharmacy, College of Medicine and Health Sciences, Wollo University, Dessie, Ethiopia; 4 Department of Health Promotion, School of Public Health, College of Medicine and Health Sciences, Wollo University, Dessie, Ethiopia

**Keywords:** Time, Postpartum, Family planning, Mother, Dessie, Ethiopia

## Abstract

**Background:**

Timing of initiation of family planning is an important determinant for the health status of the mother and her child. One-fourths of mothers in developing countries who wanted to space or limit their children were not using family planning methods at right time after delivery. Despite, the existence of many literatures about postpartum family planning, the timing of it is not yet studied. Thus, this study aimed to assess time to postpartum family planning and its predictors among mothers coming for first measles vaccination in Dessie city, Northeast Ethiopia.

**Methods:**

An institutional-based retrospective follow-up study was conducted among mothers coming for infant vaccination at Family Guidance Association of Ethiopia, Dessie Model Clinic in Dessie City. A systematic sampling technique was used. The data were entered and analyzed using Epi Data version 3.1 and STATA version 14.0, respectively. Kaplan–Meier and Cox regression model were used to test the time and predictors of postpartum family planning initiation. Adjusted hazard ratio with 95% CI was used to test the strength of association at a p-value of 0.05.

**Results:**

The rate of postpartum FP initiation was 0.6% with 95% CI  (0.0056, 0.0069). Keeping the effect of confounder constant, age of the women 20–24 [AHR = 2.63, 95% CI (1.65,4.19)], 25–29 [AHR = 3.66, 95% CI (2.35,5.73)], 30–34 [AHR = 2.79, 95% CI (1.75,4.46)], getting family planning counseling [AHR = 1.78, 95% CI (1.26, 2.52)], want more child [AHR = 0.47, 95% CI (0.34, 0.66)], having history of abortion [AHR = 0.54, 95% CI (0.36,0.81)] and wanted last pregnancy [AHR = 0.69, 95% CI (0.49, 0.97) were significantly associated with postpartum family planning initiation.

**Conclusions:**

Age, history of abortion, counseling about family planning, the status of last pregnancy and want more child were significantly associated with postpartum family planning use. Continuous emphasis should be given for health care providers to encourage their counseling services for their customers at different age groups with special attention given for elders.

**Supplementary Information:**

The online version contains supplementary material available at 10.1186/s12978-023-01608-w.

## Introduction

Postpartum Family Planning (PPFP) is defined as the use of contraceptive methods in the first twelve months following childbirth to prevent unintended and closely spaced pregnancies [1, 2]. World Health Organization (WHO) recommends at least twenty four months and six months interval after a live birth and miscarriage/induced abortion respectively in order to reduce the risk of adverse maternal, prenatal and infant outcomes [3]. It is also recommended that postpartum women should initiate family planning within the first six weeks of the postpartum period [4].

The median survival time for postpartum initiation of family planning was 120 days as reported from previous study conducted in Southern Ethiopia [5]. In addition, the median survival time for initiation of postpartum FP was 210 and 570 days as shown from studies done in Tanzania and Uganda respectively [6, 7].

Timing of family planning initiation is an important determinant for the health and survival of the newborn and the mother. Worldwide, more than 90% of women during the first year of the postpartum period want to either delay or avoid future pregnancies, however, in most cases, sexual activity is resumed without using any contraceptive method [8]. Globally 12% of women in the reproductive age group who need to avoid pregnancy during the postpartum period are not using family planning [9]. Demography and Health Survey (DHS) analysis report of 27 developing countries also showed that more than 65% of women who want to avoid pregnancies for at least 2 years are not using any form of contraceptive [10]. An analysis of data from the Demographic and Health Survey of 17 developing countries also found that an average of 25% of couples who wanted to space or limit their children were not using any form of modern contraceptive method at the right time after delivery [11].

Pregnancies that occurred in the first year of the postpartum period were indicated to be linked with increased risk of maternal morbidity and mortality, preterm birth, low birth weight, small for gestational age (SGA), puerperal sepsis, premature rupture of membranes, and anemia [12, 13]. Women who have unintended births also tend to suffer from reduction in overall physical wellbeing, postpartum depression, feelings of powerlessness, and increased time pressures [14].

Studies revealed that age [15–18], maternal educational status [5, 7, 15–17, 19–27], marital status [17–19, 27, 28],employment status [18, 22, 24, 28],wealth status [15, 16, 29], husband education status [29, 30], discussion with partner [21, 22, 24, 25, 31–33], knowledge on contraceptive methods [21, 25, 26, 33–35], family planning counseling [23, 33, 36–38], resumption of sex [22, 23, 33, 34, 37–39], menses resumption [18, 23, 25, 26, 28, 31, 33, 37, 39–41], antenatal care service utilization [25, 27, 31, 37, 41, 42],postnatal care utilization [23, 26, 29–31, 35, 42], and history contraceptive use [18, 22, 24, 28, 32, 33] were factors associated with postpartum family planning utilization.

Improving maternal and child health, and family planning utilization are key components of targets 3.1 and 3.7 of sustainable development goals (SDGs) [43]. The government of Ethiopia has also developed, adopted, and ratified policies, strategies, and guidelines to decrease unmet need for family planning to improve maternal and child health [44–47]. Additionally, a significant number of civil society and non-profit organizations are also making investments to address the low utilization of postpartum family planning in different parts of the country. However, postpartum family planning utilization is still low [48].

Determining the time to postpartum family planning initiation and its predictor is crucial to reduce the problem. Previous studies conducted on postpartum family planning utilization did not consider the timing of postpartum family planning initiation and its predictors [26–31, 34–37, 39, 41]. Some studies used logistic regression model by categorizing survival data. However, the appropriate model for survival data is cox-regression model which enables to determine predictors with the account censoring data. Therefore, this study aimed to determine time to postpartum family planning and its predictors among mothers coming for first measles vaccination at Family Guidance Association of Ethiopia, Dessie Model Clinic, in Dessie city, Northeast Ethiopia.

## Methods and materials

### Study setting, study design and population

The study was conducted in Dessie city Family Guidance Association of Ethiopia, Dessie Model Clinic for 23 days from May 1–September 30, 2021. The study participants were followed retrospectively from delivery until they came for infant first measles vaccination. Dessie is the center of South Wollo Zone located 401 km away from Addis Ababa, the capital city of Ethiopia, and 480 km away from Bahir Dar [49]. An institution-based retrospective follow up study design was conducted. We have taken the follow-up period starting from birth up to the first measles vaccination by asking the date of delivery for the index child. It was historical cohort. All mothers coming for infant first measles vaccination at FGAE, Dessie Model Clinic during the study period were included in this study.

### Sample size determination and sampling procedure

The sample size was determined by using double population proportion formula by taking the percent of outcome among unexposed (17.5%) from previous study conducted in Southern Ethiopia [31]. By taking a 10% non-response rate, the final sample size was 491.

Systematic random sampling was employed to select samples from the study population. The average number of mothers coming for first measles vaccination at FGAE Dessie Model Clinic in one month is 996. K was calculated by dividing the average patient flow per month to the total sample size and that was two. The random start was selected by using lottery method and became two. The study participants were selected every two intervals.

### Variable measurement

The dependent variable was time to postpartum family planning (in days). Women who initiate family planning from the date of delivery until they come for infant first measles vaccination were taken as an event. Similarly, women who did not initiate family planning until they come for first infant measles vaccination were considered as censored.

*Utilization of PPFP*: If a woman is using any one of the following modern contraceptive methods within 12 months following her recent childbirth: pill, intrauterine device, injectable, condom (men or women), women sterilization, implants, and Lactational amenorrhea method [22, 25].

### Data collection procedure and quality control

The data were collected using pretested structured Amharic version questionnaire adapted from previous studies [5, 7, 20, 31]. Eight data collectors collected the data, and two supervisors made supervision. The data collectors and supervisors were Midwives. Two qualified translators were also recruited. The questionnaire was developed in English, then translated to Amharic language, and again translated back to English to ensure consistency. Data collectors and supervisors were trained for two days on the objective of the study, the content of the questionnaire, and the data collection procedure. Data were pretested on 5% of the total sample size in the area other than the study area that is Dessie health center and based on feedback obtained from the pretest, the necessary modification was performed. During the study period, the collected data were checked continuously on daily basis for completeness.

## Data processing and analysis

The data were entered into Epi Data version 3.1 and exported to Stata/SE version 14.0 for analysis. Tables, graphs, charts, and texts were used to present descriptive data. To compare survival curves or to estimate time to postpartum FP initiation Kaplan–Meier survival estimate was used. Cox regression (proportional hazards) model was used to test the presence of association between independent variables and time to postpartum family planning initiation. In the analysis, first Bivariable cox-regression model was fitted and variables eligible for multivariable analysis were selected to multivariable cox-regression. Adjusted hazard ratio with 95% CI was used to test the strength of association at a p-value of 0.05. Cox proportional hazard assumption was checked and satisfied for all predictors.

$$\text{h}(\text{t};\text{x}_{\text{i}} ) = \text{h}_{0} (\text{t})\exp (\text{x}\beta_{i} )$$

The cox-proportional hazards model gives an expression for the hazard at time t for an individual with a given specification of a set of independent variables denoted by 'x', which are predictor variables that are being modeled to predict the hazard of an individual. The Cox proportional-hazards regression assumes the relationship for one covariate where h0(t) is the baseline hazard function, xi are the covariates and βi are the coefficients.

## Results

### Socio-demographic characteristics of the women

Four hundred and seventy-two women were participated, making the response rate 96.13%. Of the total participants, 240 (50.85%) were in the age group 25–29. From all respondents, only nine (19.10%) participants resided in the rural area and 462 (97.88%) were married. Looking at educational status 26 (5.51%) of women had no education. Three hundred twenty-four (68.64%) of participants were housewives and 259 (54.87%) were Muslim. When we see the educational status and occupation of women's husbands 201 (43.51%) had tertiary education and 163 (35.28) were government employees respectively (Table 1).

**Table 1:** Socio-demographic characteristics of women coming for infants first measles vaccination at Family Guidance Association of Ethiopia, Dessie Model Clinic, Northeast Ethiopia, 2021 (n = 472)

Variables	Category	Frequency (%)
Age	15–24	83 (17.58)
	25–29	240 (50.85)
	30–34	89 ( 18.86)
	35–49	60 (12.71)
Residence	Rural	9 (1.91)
	Urban	463 (98.09)
Marital status	Not married	10 (2.12)
	Married	462 (97.88)
Educational level	No education	26 (5.51)
	Primary	139 (29.45)
	Secondary	130 (27.54)
	Tertiary	177 (37.50)
Religion	Orthodox	203 (43.01)
	Muslim	259 (54.87)
	Others	10 (2.12)
Occupation	Governmental employee	90 (19.07)
	Merchant	36 ( 7.63)
	Private employee	22 (4.66)
	Housewife	324 (68.64)
Husband Educational level	No education	34 (7.36)
	Primary	64 (13.85)
	Secondary	163 (35.28)
	Tertiary	201 (43.51)
Husband Occupation	Governmental employee	163 (35.28)
	Merchant	126 (27.27)
	Private employee	94 (20.35)
	Daily labor	53 (11.47)
	Driver	17 (3.68)
	Student	9 (1.95)
Average monthly income	0–5000 ETB	172 (36.44)
	5001–8000 ETB	75 (15.89)
	8001–14,000 ETB	110 (23.31)
	14,001–53,000 ETB	115 (4.36)

ETB: Ethiopian Birr

### Behavioral and psychosocial factors

Of the total respondents, 154 (32.63%) drink alcohol, 92 (19.49) chew chat and none of the respondents smoke cigarette. Four hundred fifty-three (95.97%) respondents discuss about FP with their husbands and 447 (94.70%) had support from their husbands to use FP. In addition 121 (25.64%) and 416 (88.14%) women got family planning training and counseling respectively. From the total participants, 401 (84.96%) women got information about FP from health providers and 422 (89.41%) women were exposed to media (Table 2).

**Table 2:** Behavioral, and psychosocial characteristics of women coming for infant first measles vaccination at Family Guidance Association of Ethiopia, Dessie Model Clinic, Northeast Ethiopia, 2021 (n = 472)

Variable	Category	Frequency (%)
Alcohol drinking	No	318 (67.37)
	Yes	154 (32.63)
Chat chewing	No	380 (80.51)
	Yes	92 (19.49)
Discussion with husband about FP	No	19 (4.03)
	Yes	453 (95.97)
Husband support to use FP	No	25 (5.3)
	Yes	447 (94.70)
Getting training on FP	No	351 (74.36)
	Yes	121 (25.64)
Getting counseling on FP	No	56 (11.86)
	Yes	416 (88.14)
Frequency of sexual contact	At least three per week	261 (55.30)
	Three times per month	179 (37.92)
	Three times per year	32 (6.78)
Source of information for FP	Provider	401 (84.96)
	Family	7 (1.48)
	Friend	22 (4.66)
	Media	42 (8.90)
Media exposure	Exposed	422 (89.41)
	Not exposed	50 (10.59)

FP: family planning

### Gynecologic and obstetric factors

From the total participants, 361 (76.48%) women had 1–2 number of live children and 288 (61.02%) had desired number of children 3–4. Forty-six (9.75%) and 132 (27.97%) women had history of abortion and CS respectively. All of the respondents hear about contraceptives and had history of antenatal care utilization and institutional delivery. In addition, 410 (86.86%) women's last pregnancy was wanted and 144 (30.51%) women had postnatal care utilization. Four hundred respondents (84.75) want to have more children (Table 3).

**Table 3:** Gynecologic and obstetric characteristics of women coming for infant first measles vaccination at Family Guidance Association of Ethiopia, Dessie Model Clinic, Northeast Ethiopia, 2021 (n = 472)

Variable	Category	Frequency (%)
No of alive children	1–2	361 (76.48)
	3–4	97 (20.55)
	≥ 5	14 (2.97)
Desired number of children	1–2	42 (8.90)
	3–4	288 (61.02)
	≥ 5	142 (30.08)
Want more child	No	72 (15.25)
	Yes	400 (84.750
History of abortion	No	426 (90.25)
	Yes	46 (9.75)
History of cesarean section	No	340 (72.03)
	Yes	132 (27.97)
Status of last pregnancy	Unintended	62 (13.14)
	Wanted	410 (86.86)
History of dead child	No	436 (92.37)
	Yes	36 (7.63)
Postnatal care utilization	No	328 (69.49)
	Yes	144 (30.51)
Status of menses	No	84 (17.80)
	Yes	388 (82.20)

### Family planning utilization

From the total participants, 416 (88.14%) women used contraceptive in their lifetime and 370 (78.39%) women were using contraceptive currently. From the FP users 357 (96.49%) choose the type of method by themselves and 295 (79.73%) use FP for spacing. Looking at the type of FP used, 48.38% of women use injectable and 6.76 used IUCD (Intrauterine Contraceptive Device).

### Time to postpartum family planning initiation

**Fig. 1:**
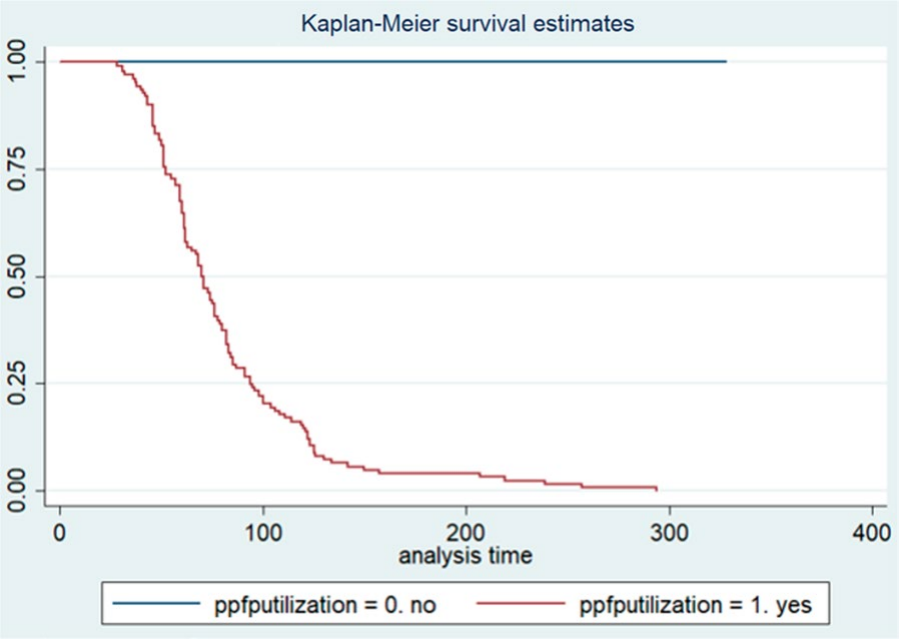
Kaplan Meier estimate for postpartum FP initiation of women coming for infant first measles vaccination at Family Guidance Association of Ethiopia, Dessie Model Clinic, Northeast Ethiopia, 2021

In this study, the participants were assessed for 59,308 person-days retrospectively. The median survival time and the Inter Quartile Range for postpartum initiation of family planning was 82 and 98 (59, 157) days respectively. The minimum and the maximum days for FP initiation were 28 and 328 days respectively. The rate of postpartum FP initiation per day was 0.6 with 95% CI (0.56, 0.69) (Fig. 1).

### Factors associated with time to postpartum FP initiation

After adjusting for confounding variables the Cox proportional hazards model shows that age of the respondent, FP counseling, history of abortion, history of dead child, want more child, frequency of sexual intercourse, and status of last pregnancy had a significant association with postpartum family planning initiation.

Keeping the effect of other variables constant, the rate of postpartum family planning initiation of women at any given time decreases by 7% for a unit increase in age [AHR = 0.93, 95% CI (0.91, 0.96)]. The rate of postpartum family planning initiation of women at any given time increases by 1.91 times among those women who get counseling about FP as compared to women who do not get counseling about FP [AHR = 1.91, 95% CI (1.34, 2.73)]. Those women who have history of abortion had 42% lower rate of postpartum FP initiation as compared to women who have no history of abortion [AHR = 0.58, 95% CI (0.37,0.92)]. Women who want more children had 50% less likely to initiate postpartum FP as compared to their counterparts [AHR = 0.50, 95% CI (0.37, 0.92)]. Similarly, those women whose last child was unintended were 1.74 times more likely to have postpartum FP initiation [AHR = 1.74, 95% CI (1.24, 2.42). Women who had history of dead children have 57% less likely to utilize postpartum FP as compared to women who had no history of dead child [AHR = 0.43, 95% CI (0.26, 0.72). Those participants who practice sexual intercourse at least three times per week had 2.53 times more likely to use postpartum FP as compared to women who practice three times per year [AHR = 2.53, 95% CI (1.53, 4.20). Similarly, women who practice sexual intercourse three times per month had 1.97 times more likely to use postpartum FP as compared to women who practice three times per year [AHR = 1.97, 95% CI (1.18, 3.28) (Table 4).

**Table 4:** Cox proportional hazards model for factors associated with women coming for infant vaccination at Family Guidance Association of Ethiopia, Dessie Model Clinic, Northeast Ethiopia, 2021

	Category	CHR (95% CI) n = 472	AHR (95% CI) n = 472
Age	**–**	0.96 (0.95, 0.99)	0.93 (0.91, 0.96)*
Occupation	Housewife	1	1
	Merchant	1.02 (0.69, 1.48)	1.39 (0.89, 2.15)
	Private employee	1.49 (0.94, 2.38)	1.28 (0.77, 2.11)
	Government employee	0.93 (0.71, 1.21)	1.12 (0.83, 1.50)
Chewing chat	No	1	1
	Yes	0.94 (0.78, 2.23)	1.21 (0.92,1.59)
Drinking alcohol	No	1	1
	Yes	1.28 (0.74, 2.23)	1.14 (0.87, 1.49)
PNC utilization	No	1	1
	Yes	0.68 (0.54, 0.86)	0.90 (0.47,1.71)
Training about FP	No	1	1
	Yes	0.82 (.64, 1.04)	0.87 (0.66, 1.15)
FP counseling	No	1	1
	Yes	1.62 (1.15, 2.28)	1.92 (1.34, 2.73)*
History of abortion	No	1	1
	Yes	0.52 (0.35, 0.76)	0.58 (0.38, 0.92)*
Media exposure	Not exposed	1	1
	Exposed	0.74 (0.52, 1.05)	0.81 (0.54,1.18)
Want more child	No	1	1
	Yes	0.68 (0.52, 0.90)	0.50 (0.36, 0.70)*
History of dead child	No	1	1
	Yes	0.45 (0.28, 0.71)	0.43 (0.25, 0.72)*
Number of live born	1–2	1.96 (0.92, 4.15)	1.45 (0.54, 3.86)
	3–4	3.01 (1.39, 6.53)	2.24 (0.88, 5.71)
	> = 5	1	1
Status of last pregnancy	Unintended	1.52 (1.14, 2.03)	1.74 (1.24, 2.42)*
	Wanted	1	1
Frequency of sex	At least three per week	1.66 (1.05, 2.63)	2.53 (1.53, 4.20)*
	Three per month	1.26 (0.79, 2.03)	1.97 (1.18, 3.28)*
	Three per year	1	1

PNC: Postnatal care; CHR: Crude Hazard Ratio

*Statistically significant (P-value less than 0.05), AHR: Adjusted hazard ratio and FP: Family planning

## Discussion

The rate of postpartum FP initiation per day was 0.6% with 95% CI = (0.56, 0.69). Keeping the effect of confounders constant, age, the status of last pregnancy, want more child, history of abortion, and family planning counseling were significantly associated with postpartum FP initiation.

In this study, the median survival time for postpartum initiation of family planning was 82 days. This finding was lower than a previous study done in Ethiopia, which reported a median time to postpartum FP initiation of 120 days [5]. But it was lower than the median survival time for initiation of FP to studies done in Tanzania and Uganda which were 210 and 570 days, respectively [6, 7]. This may be due to socio-cultural differences between countries and the variation in the study time, which have more than 5 years difference. Moreover, 61.6% of women from study conducted in Tanzania and 86.3% of women included in study conducted in Uganda were from rural area.

The result of this study reveals that younger age women were more likely to initiate postpartum FP. This is in line with studies done in Debre Tabor [18], Nigeria [19], Uganda [15], and Kenya [17]. The reason for this might be younger women are more fertile and perhaps are more motivated to initiate contraception earlier because they do not want to interrupt their education or other pursuits or women in the higher age groups may assume that they are not fecund and therefore, have no need for contraception.

In this study, mothers who desire to have a lower number of children were more likely to use PPFP. This finding is consistent with a study done in Western Ethiopia [42] and Northern Nigeria [50]. The reason behind this is clear that those women who desired to have more children will not need to use contraception.

The finding of this study revealed that those women who have got counseling about FP were more likely to use postpartum FP. There was also a similar report from studies done in Bahir Dar [36], Gondar [41], Western Ghana [33], Mexico [51], and India [52]. This might be due to the reason that when health professionals discuss about birth preparedness and complication readiness plan they are more likely to discuss the danger signs of pregnancy and the probability of their occurrence in closely spaced pregnancy; which might influence the decision of women to use postpartum family planning.

Those women whose last pregnancy was unintended were more likely to initiate postpartum Fp. This result is contrary to a study conducted in Arba-Minch town, Ethiopia [27]. The possible explanation for this might be women who faced unintended pregnancy may become cautious for the future in order not to repeat the previous mistake.

Women who have history of abortion and history of dead child were less likely to use postpartum family planning. The possible justification might be that they will want to replace the lost child. In addition, if they had previous history of family planning utilization before they faced such problems they may wrongly perceive that family planning may be the cause of that problem.

Women who resumed sexual intercourse after delivery were more likely to initiate postpartum FP. This result is consistent with studies done in Debre-Birhan [39], Aksum [34], Ghana [33], and Malawi [38]. The reason for this could be that women who practice sexual intercourse have the possibility to have sex during the period in which fertility will occur.

The practical implication of this study will be those women who initiated postpartum family planning utilization in the early postpartum period will be better able to prevent unintended pregnancy and subsequent abortion and other complications related to it.

In spite of its strength, this study has limitations. Since the study was retrospective, the reliability of this study depends on the mother’s recall of past events regarding the different information and therefore may be subject to recall bias. In addition, since it was done in one health institution it may not be generalizable to the general population.

## Conclusions

The rate of postpartum family planning initiation of women coming for infant vaccination at Family Guidance Association of Ethiopia, Dessie Model Clinic was late since timely initiation of postpartum family planning initiation is defined as the initiation of FP within the first six weeks from delivery. Age of the respondent, counseling about FP, want more child, the status of last-child, history of abortion, history of dead child, and frequency of sex have a significant association with postpartum FP initiation.

Continuous emphasis should be given for health care providers to encourage their counseling services for their customer at different age group with especial attention given for elders. Health care providers should be given proper direction to increase and sustain utilization of postpartum FP in all health services delivery points. Moreover, younger people should be motivated to keep up the utilization of Postpartum FP and to advice or share their awareness for those who are not using FP. In addition, give advice to those old aged women to use FP by giving information about the possibility of fertility if their menstruation is coming. Further prospective cohort study is needed to point out the specific time of initiation of postpartum family planning for respective women. In addition, community based research is better to be conducted to address those women who did not come for vaccination.

## Supplementary Information

**Additional file 1**. S2: Data set used for this study.

## Data Availability

All the data used and analyzed during this study are attached with the manuscript as supporting information (Additional file 1).

## Abbreviations

AHR: Adjusted hazard ratio
BSc: Bachelor of Science
CI: Confidence interval
DHS: Demographic and Health Survey
EDHS: Ethiopian Demographic and Health Survey
FGAE: Family Guidance Association of Ethiopia
FMOH: Federal Ministry of Health
FP: Family planning
PPFP: Postpartum family planning
SDGs: Sustainable development goals
SGA: Small for gestational age
WHO: World Health Organization
